# Genistein Enhances the Radiosensitivity of Breast Cancer Cells via G_2_/M Cell Cycle Arrest and Apoptosis 

**DOI:** 10.3390/molecules181113200

**Published:** 2013-10-24

**Authors:** Xiongxiong Liu, Chao Sun, Xiaodong Jin, Ping Li, Fei Ye, Ting Zhao, Li Gong, Qiang Li

**Affiliations:** 1 Institute of Modern Physics, Chinese Academy of Sciences, Lanzhou 730000, Gansu, China; 2 Key Laboratory of Heavy Ion Radiation Biology and Medicine, Chinese Academy of Sciences, Lanzhou 730000, Gansu, China; 3 University of Chinese Academy of Sciences, Beijing 100049, China

**Keywords:** breast cancer cells, radiosensitivity, genistein, G_2_/M arrest, apoptosis

## Abstract

The aim of the present study was to investigate the radiosensitizing effect of genistein, and the corresponding mechanisms of action on breast cancer cells with different estrogen receptor (ER) status. Human breast cancer cell lines such as MCF-7 (ER-positive, harboring wild-type p53) and MDA-MB-231 (ER-negative, harboring mutant p53) were irradiated with X-rays in the presence or absence of genistein. Cell survival, DNA damage and repair, cell cycle distribution, cell apoptosis, expression of proteins related to G_2_/M cell cycle checkpoint and apoptosis were measured with colony formation assays, immunohistochemistry, flow cytometry and western blot analysis, respectively. Genistein showed relatively weak toxicity to both cell lines at concentrations in the range of 5–20 μM. Using the dosage of 10 μM genistein, the sensitizer enhancement ratios after exposure to X-rays at a 10% cell survival (IC_10_) were 1.43 for MCF-7 and 1.36 for MDA-MB-231 cells, respectively. Significantly increased DNA damages, arrested cells at G_2_/M phase, decreased homologous recombination repair protein Rad51 foci formation and enhanced apoptotic rates were observed in both cell lines treated by genistein combined with X-rays compared with the irradiation alone. The combined treatment obviously up-regulated the phosphorylation of ATM, Chk2, Cdc25c and Cdc2, leading to permanent G_2_/M phase arrest, and up-regulated Bax and p73, down-regulated Bcl-2, finally induced mitochondria-mediated apoptosis in both cell lines. These results suggest that genistein induces G_2_/M arrest by the activation of the ATM/Chk2/Cdc25C/Cdc2 checkpoint pathway and ultimately enhances the radiosensitivity of both ER+ and ER- breast cancer cells through a mitochondria-mediated apoptosis pathway.

## 1. Introduction

Female breast cancer is the most common disease in women. About 1,200,000 women suffer and 500,000 deaths per year occur from breast cancers worldwide [1,2]. Furthermore, the incidence of breast cancers is expected to rise in the coming years and the age of patients is tending to be younger [3]. Clinical tests for the presence or absence of estrogen receptor (ER) are routinely run to facilitate the choice of therapy, with human breast cancer cells currently being categorized into three broad classes based on the level of ER presented in the cells and on the functional state of ER [4]. In class 1, breast cancer cells (such as MCF-7) are ER-positive, hormone-dependent, responsive to estrogen and successfully treated with anti-hormones such as tamoxifen. In class 2, breast cancer cells (such as _21_PT) are ER-positive, hormone-independent and non-responsive to anti-hormones. Class 3 breast cancer cells (such as MDA-MB-231) are ER-negative, hormone-independent, hormone non-responsive and high-grade malignant tumors. Treatment options for breast cancer patients are limited to conventional cytotoxic chemotherapy, which is not effective against high-grade malignant tumors, and there are serious side effects [5,6,7]. Thus, to improve the local control and survival rate, radiation therapy combined with radiosensitizing reagents is one of the most effective treatments against breast cancers.

It is well known that cells have different radiosensitivities during diverse phases of the cell cycle. Cells are most sensitive to ionizing radiations during the G_2_/M phase, less sensitive during G_1_ phase, and least sensitive near the end of the S phase [8], so G_2_/M arrest is the major reason for cell death induced by anti-tumor drugs and radiosensitizing reagents. The progression of eukaryotic cells from G_2_ to M phase of the cell cycle depends on the activity of G_2_/M cell cycle checkpoints, which are biochemical pathways that ensure the orderly and timely progression and completion of critical events [9]. When DNA damages occur in cells, ATM preferentially activates/phosphorylates the checkpoint protein kinase Chk2 [10,11], and the thus activated Chk2 modulates the activity of Cdc25c via phosphorylation on an inhibitory site (Ser216), which either enables DNA repair or directs the cell to the apoptotic pathway [12]. The decision of cells to either remain in the G_2_/M or go through G_2_ into mitosis requires the activation of Cdc2 [13]. Cdc2 binds to cyclin B1 to form a complex that is activated at the onset of mitosis by dephosphorylation of the inhibitory sites on Cdc2 by functional Cdc25c [14].

**Figure 1:**
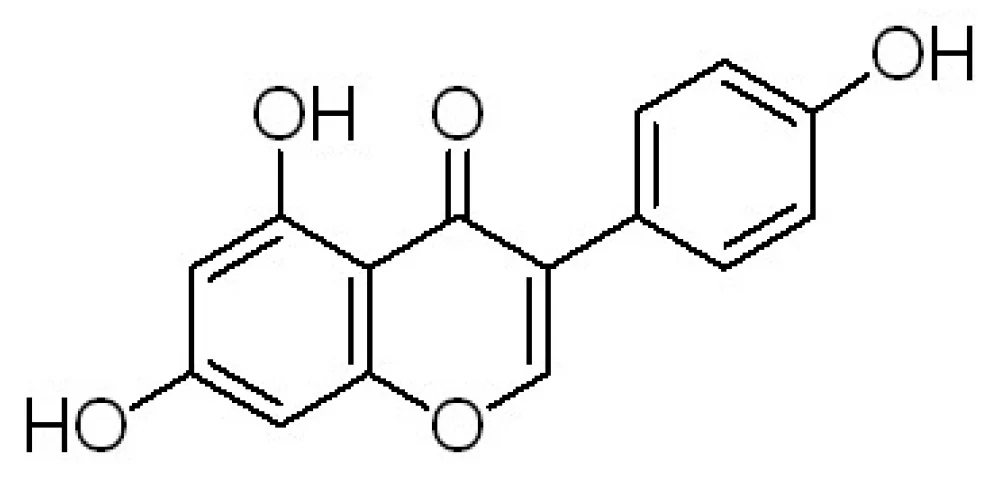
The chemical structure of genistein.

Recently, inhibition of the cell cycle and then causing apoptosis has become an appreciated target for cancer management [15]. Meanwhile, more effective radiation with minimal toxicity to the host would be of value in the treatment of cancers. Genistein (4',5,7-trihydroxyisoflavone, Figure 1) is a major isoflavone in dietary soybean and can block the proliferation of some kinds of human breast cancer cells. This effect has been attributed to a competitive inhibition by occupying ERs [16,17,18]. However, the underlying cellular and molecular mechanisms of this effect are not fully understood, especially when combined with radiation treatments. The aim of this study was to evaluate whether genistein can augment the response of both ER-positive and ER-negative human breast cancer cells to X-rays and to investigate the corresponding biological mechanisms due to involvement of genistein.

## 2. Results and Discussion

### 2.1. Effect of Genistein on Breast Cancer Cell Growth

**Figure 2:**
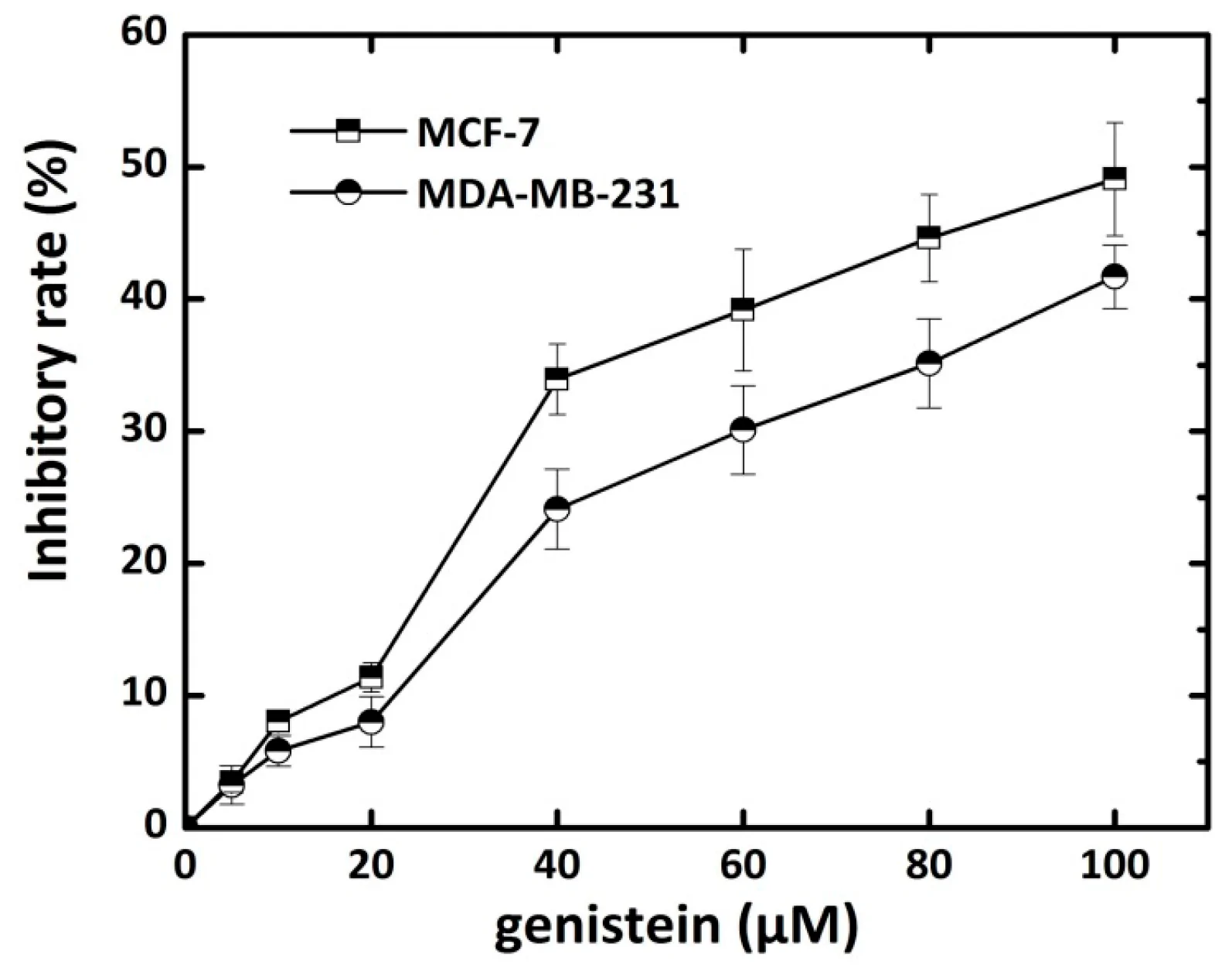
Effect of genistein on the proliferation of MCF-7 and MDA-MB-231 cells. Logarithm growth period cells were incubated with genistein at various concentrations for 48 h, then the proliferation of the cells was determined by the MTT assay. All data are presented as means ± SD from three independent experiments.

Shown in Figure 2 are the cytotoxic effects of genistein on MCF-7 and MDA-MB-231 cells, which were determined with the MTT assay after 48 h of incubation. Genistein inhibited the proliferation of the two kinds of breast cancer cells slightly when its concentration was below 20 μM. However, the inhibitory rate of genistein to both cell lines increased to more than 20% when its concentration exceeded 40 μM. Consequently genistein concentrations of 5, 10 and 20 μM were thought to be mild doses and chosen to examine its radiosensitization effects in this study.

### 2.2. Genistein Pretreatment Followed by Irradiation with X-rays Reduced the Colony Forming Ability

**Figure 3:**
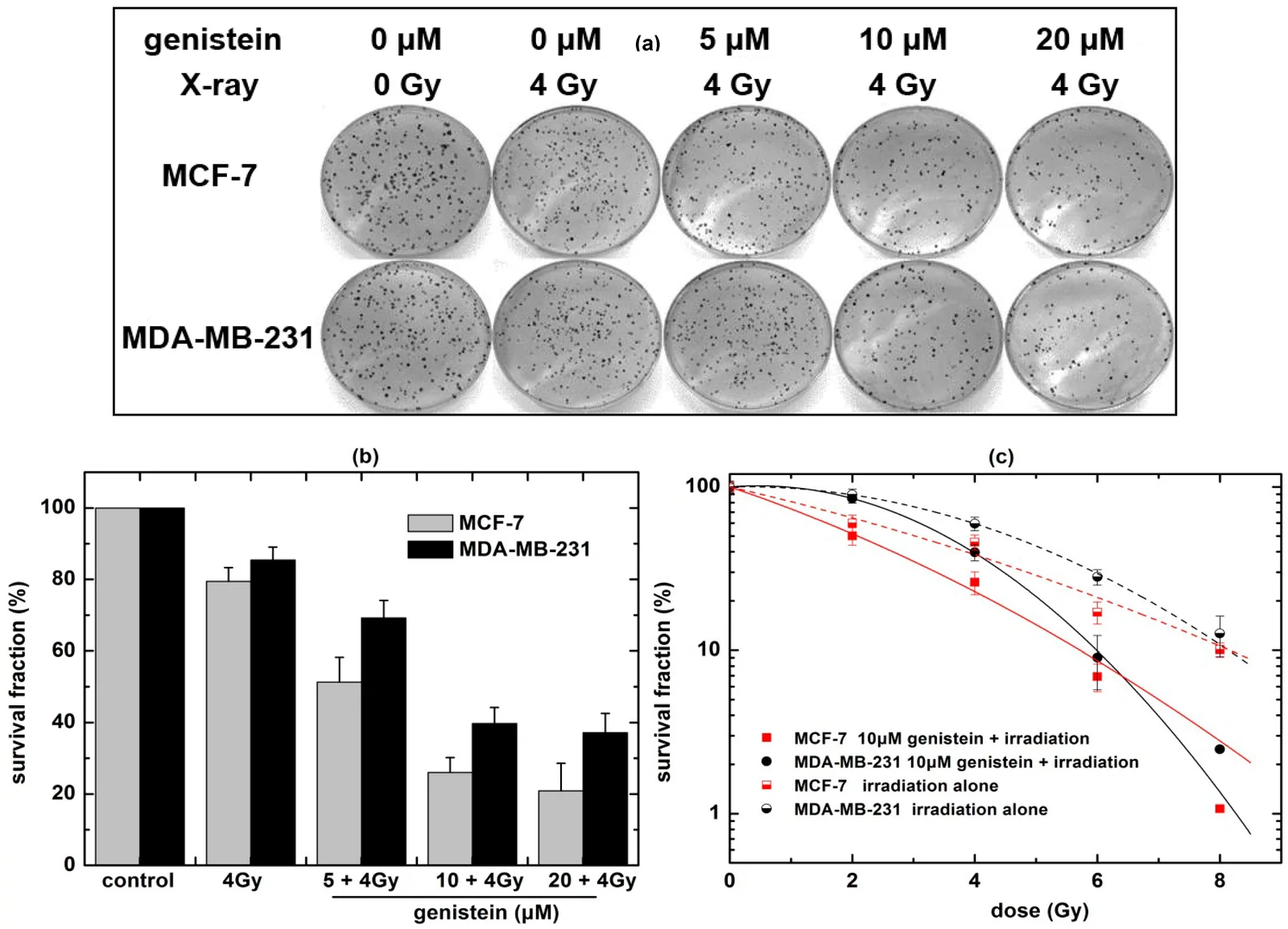
Effect of genistein on the clonogenic survival of MCF-7 and MDA-MB-231 cells. Logarithm growing period cells were pretreated with genistein for 24 h followed by X-ray irradiation. After incubation for 15 days, colonies with cells greater than 50 were counted. All data are presented as means ± SD from three independent experiments.

In order to detect the effect of different concentrations of genistein combined with X-ray irradiation on the clonogenic potential of breast cancer cells, both cell lines were irradiated with 4 Gy X-rays alone or after pretreatment with genistein (5, 10 and 20 μM) for 24 h followed by 4 Gy X-ray irradiation. Figure 3(a) shows the images of colonies and 3(b) shows the survival fraction data obtained in our experiments. For example, when the cells were pretreated with 20 μM genistein plus 4 Gy X-ray irradiation, the survival fractions of MCF-7 and MDA-MB-231 cells were decreased by 48.1 ± 0.7% and 58.2 ± 1.5%, respectively, compared those with 4 Gy X-ray irradiation alone.

To further investigate if genistein enhances the cellular sensitivity to X-rays, the breast cancer cells were exposed to 10 μM genistein for 24 h before X-ray irradiation, then subjected to the clonogenic assay. As illustrated in Figure 3(c), genistein enhanced the radiosensitivity of both cell lines with radiation enhancement ratios of 1.43 for MCF-7 and 1.36 for MDA-MB-231 cells at a 10% cell survival (IC_10_).

### 2.3. Genistein Pretreatment Followed by Irradiation with X-rays Increased DNA Damage

**Figure 4:**
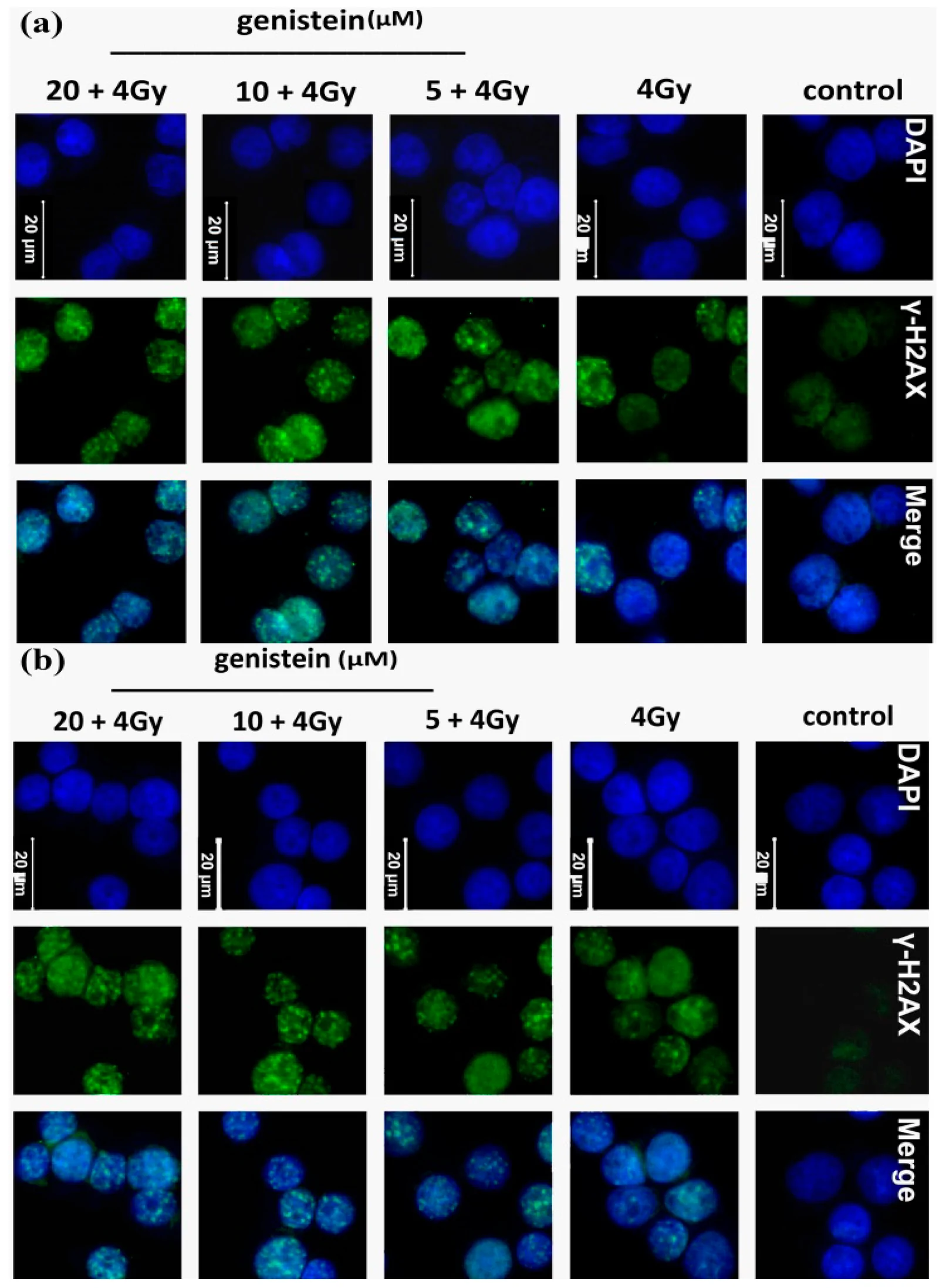
Effect of genistein combined with X-ray irradiation on the DNA damage of MCF-7 (a) and MDA-MB-231 cells (b). Nuclear staining was done with DAPI (blue) while γ-H2AX staining appeared as green points (foci). Scale bars represent 20 μm.

γ-H2AX on Ser139 is one of the key events of DNA damage response and considered to be a marker of DSB [19]. Shown in Figure 4(a) and Figure 4(b) are the results of γ-H2AX foci detection acquired in this study for MCF-7 and MDA-MB-231 cells, respectively. Clearly, when the cells were pretreated with genistein for 24 h followed by 4 Gy X-rays, at 12 h post-irradiation, the γ-H2AX foci were significantly increased in a genistein concentration-dependent manner, suggesting that DNA damage was seriously exacerbated by genistein.

### 2.4. Genistein Induced G_2_/M Phase Arrest

**Figure 5:**
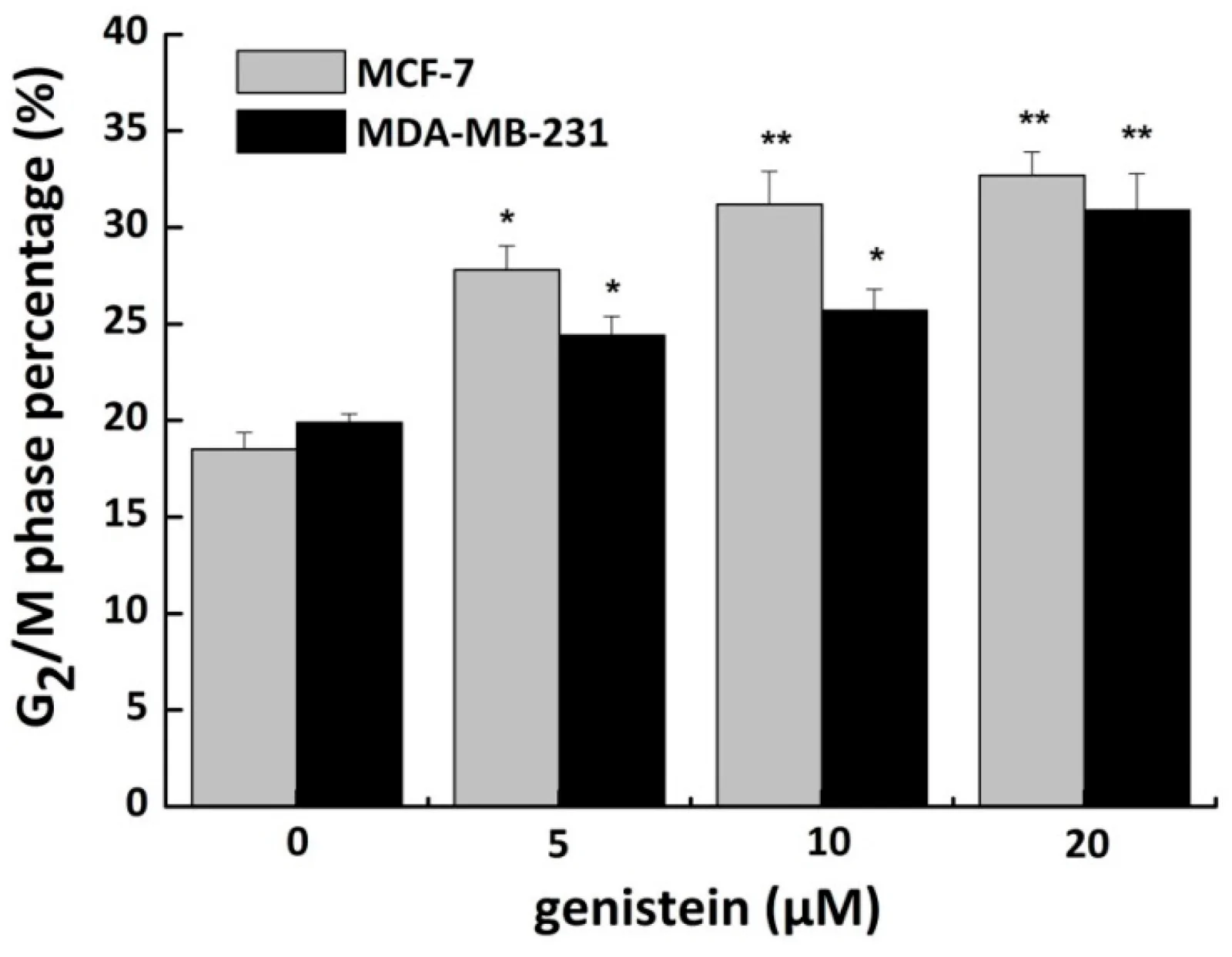
Effect of genistein on the percentage of G_2_/M phase MCF-7 and MDA-MB-231 cells. Cells were incubated with genistein (0, 5, 10 and 20 μM) for 24 h and then analyzed by flow cytometry. All data are presented as means ± SD from three independent experiments. * *p* < 0.05, ** *p* < 0.01 *versus* control group.

It is well known that cells are most sensitive to radiation during the G_2_/M phase, and G_2_/M phase arrest is one of the major reasons of cell death which induced by anti-tumor drugs and radiosensitive reagents [20]. As shown in Figure 5, when cells were pretreated with 5, 10, 20 μM genistein for 24 h, the fraction of cells at G_2_/M phase increased in a concentration-dependent manner. For example, the percentage of cells at G_2_/M phase was 32.7 ± 1.2% and 30.9 ± 1.89% in MCF-7 and MDA-MB-231 cells pretreated with 20 μM genistein, compared to 18.5 ± 0.87% and 19.9 ± 0.44% in untreated cells, respectively. Therefore, it may in part account for the effects of genistein on the enhancement of radiosensitivity of breast cancer cell lines.

### 2.5. Genistein Pretreatment Followed by Irradiation with X-rays Exacerbated G_2_/M Phase Arrest

To further prove the radiosensitizing mechanism of genistein, the influence of genistein combined with X-rays on cell cycle distribution was detected.

**Figure 6:**
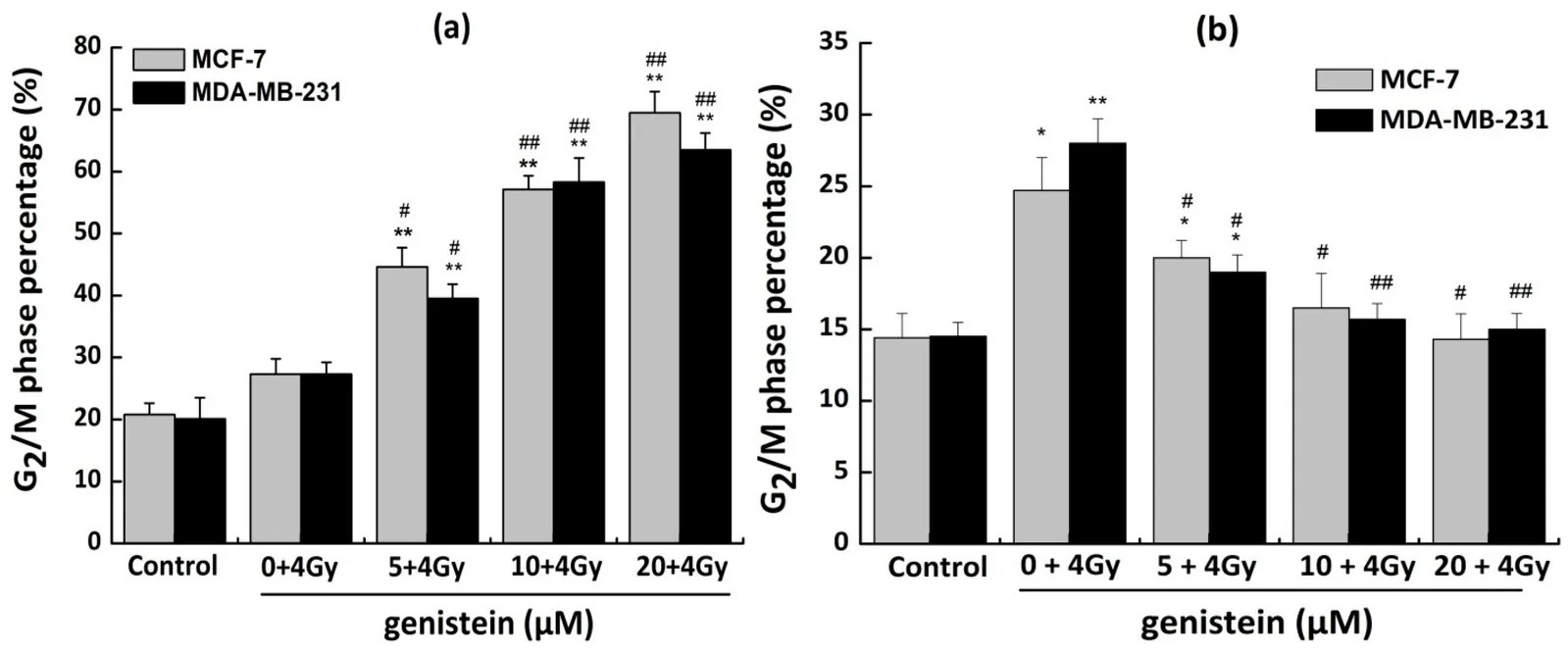
Effect of genistein combined with X-ray irradiation on the cell cycle distribution of MCF-7 and MDA-MB-231 cells. (a) G_2_/M phase percentage at 12 h post-irradiation; (b) G_2_/M phase percentage at 24 h post-irradiation. All data are presented as means ± SD from three independent experiments. * *p* < 0.05, ** *p* < 0.01 *versus* control group; ^# ^*p* < 0.05, ^## ^*p* < 0.01 *versus* X-ray irradiation alone.

As Figure 6(a) shows, genistein pretreatment exacerbated the G_2_/M arrest at 12 h post-irradiation. For example, in the 20 μM genistein pretreatment group, the percentages of MCF-7 and MDA-MB-231 cells at G_2_/M phase were increased to 69.5 ± 3.4% and 63.5 ± 2.7%, compared with 20.8 ± 1.8% and 20.1 ± 3.4% in the control groups, respectively. However, at 24 h post-irradiation (Figure 6(b)), MCF-7 and MDA-MB-231cells at G_2_/M phase were only 14.3 ± 1.9% and 15 ± 2.0% in the 20 μM genistein pretreatment group. That is to say, as the time following exposure progressed, the fraction of cells in G_2_/M phase was sharply decreased.

### 2.6. Genistein Pretreatment Followed by Irradiation with X-rays Inhibited DNA Repair and Increased Cell Apoptosis

**Figure 7:**
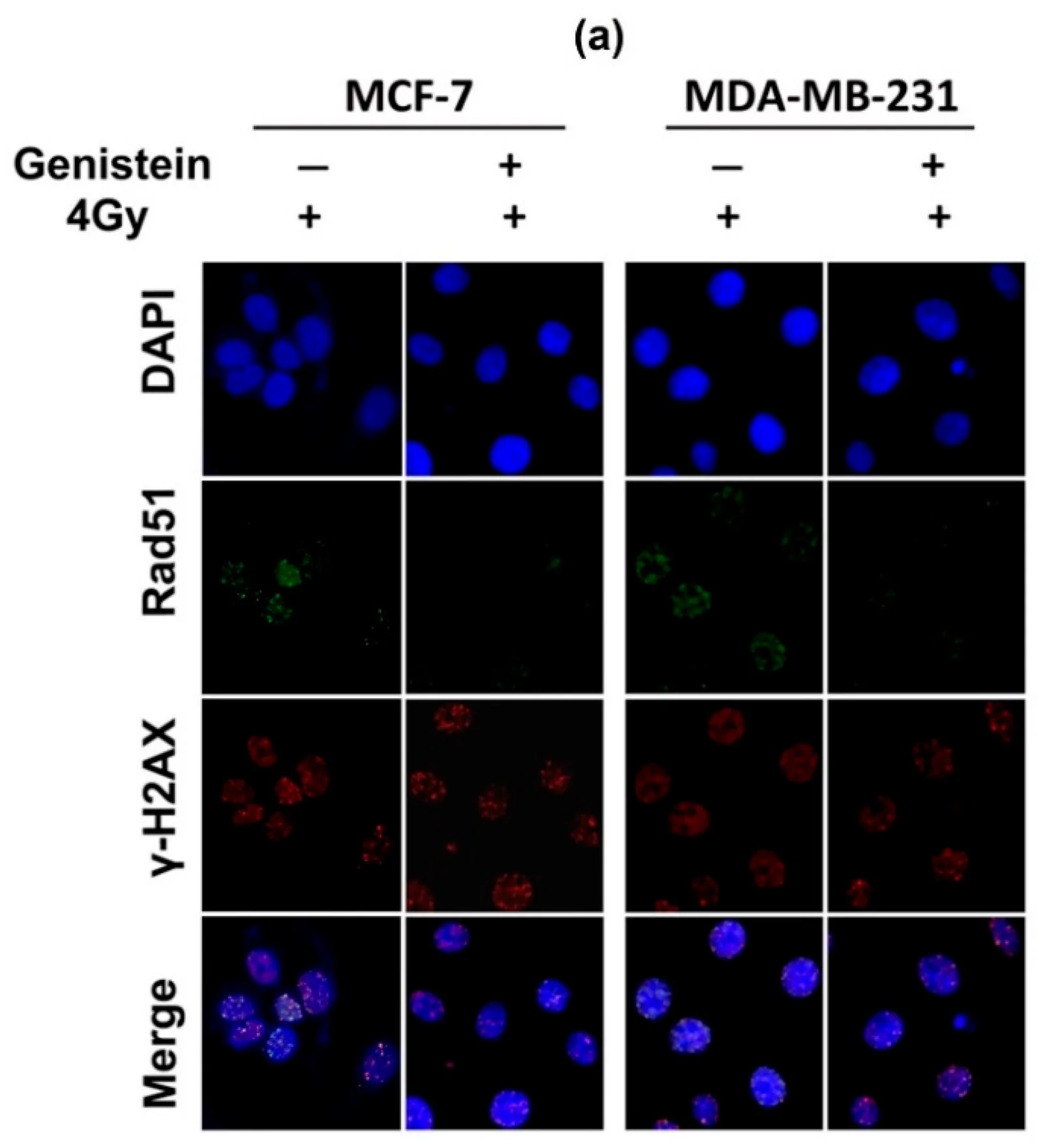

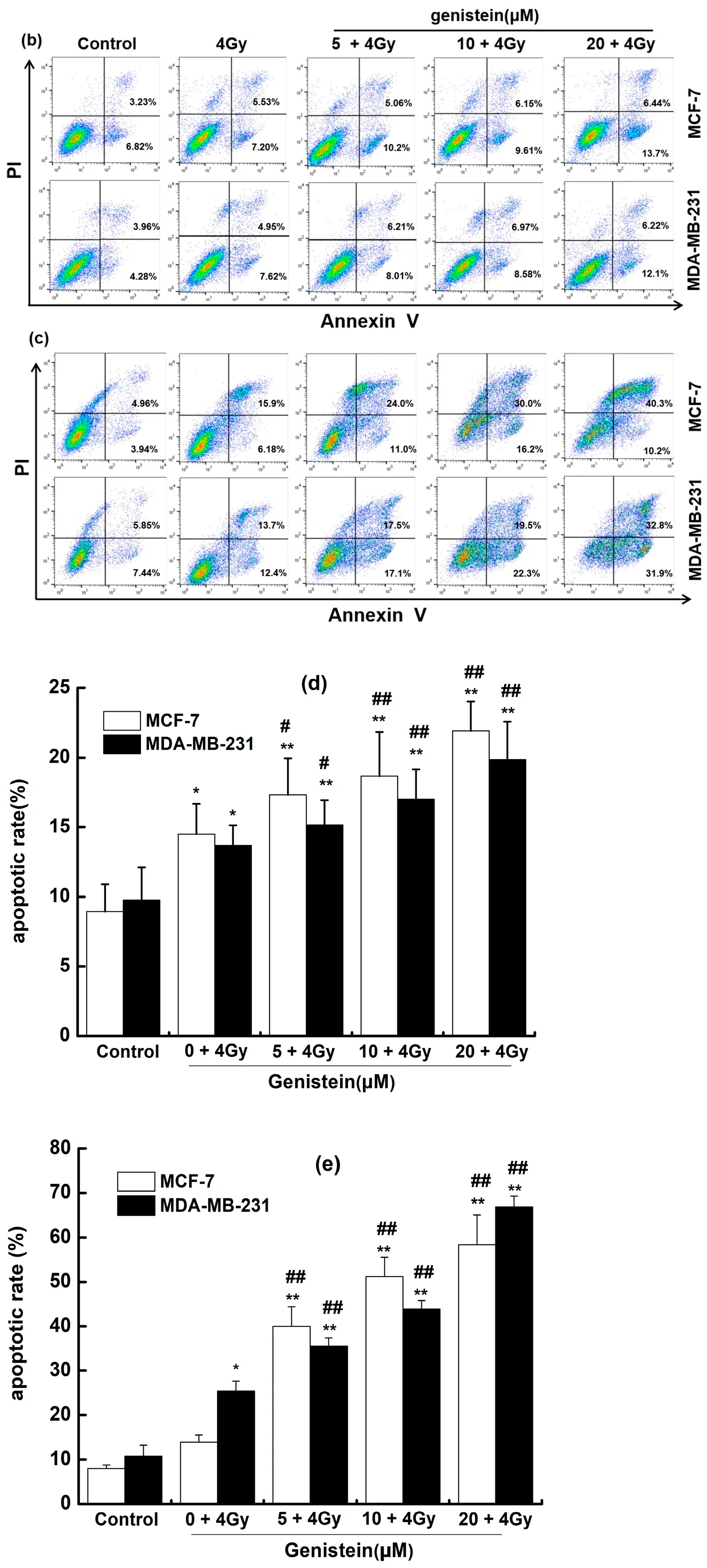
Effect of genistein combined with X-ray irradiation on the cell repair system and apoptosis of MCF-7 and MDA-MB-231 cells. (**a**) Co-localization of Rad51 (green points) and γ-H2AX (red points) foci; nuclear staining was done with DAPI (blue). Scale bars represent 20 μm; (**b**) Representative cell apoptosis of three independent experiments at 12 h post-irradiation; (**c**) Representative cell apoptosis of three independent experiments at 24 h post-irradiation; (**d**) Cell apoptotic rates at 12 h post-irradiation; (**e**) Cell apoptotic rates at 24 h post-irradiation. All data are presented as means ± SD from three independent experiments. * *p* < 0.05, ** *p* < 0.01 versus control group; ^#^ *p* < 0.05, ^##^ *p* < 0.01 versus X-rays alone.

DNA damage-induced Rad51 foci are thought to reflect repair of DNA double-strand breaks by homologous recombination; they represent the level of the DNA repair system. The co-localization of γ-H2AX and Rad51 foci is shown in Figure 7(a). Compared with the group of irradiation alone, cell pretreatment with 10 μM genistein followed by 4Gy X-ray irradiation inhibited the formation of Rad51 foci in both MCF-7 and MDA-MB-231 cells, but the γ-H2AX foci continued. These data proved that disturbance of DNA homologous recombination repair by genistein might be the major cause impairing DNA repair in cells at G_2_/M phase.

Next, we investigated whether genistein enhancement of the radiosensitivity of breast cancer cells was associated with cell apoptosis. Cells were pretreated with a range of genistein concentrations for 24 h, followed by 4 Gy X-rays. Figure 7b,c show the representative apoptosis results at 12 h and 24 h post-irradiation. At 12 h post-irradiation, the apoptotic rates were 21.9 ± 2.1% and 19.9 ± 2.7% in MCF-7 and MDA-MB-231 cells in the 20 μM genistein pretreatment group, in contrast to 8.9 ± 2.0% and 9.8 ± 2.4% in the control groups, respectively (Figure 7d). At 24 h post-irradiation, the apoptotic rate increased more significantly (Figure 7e).

### 2.7. Genistein Pretreatment Followed by Irradiation with X-rays Activated G_2_/M Checkpoint Proteins and Affected the Expression of Cell Apoptosis Associated Proteins

**Figure 8:**
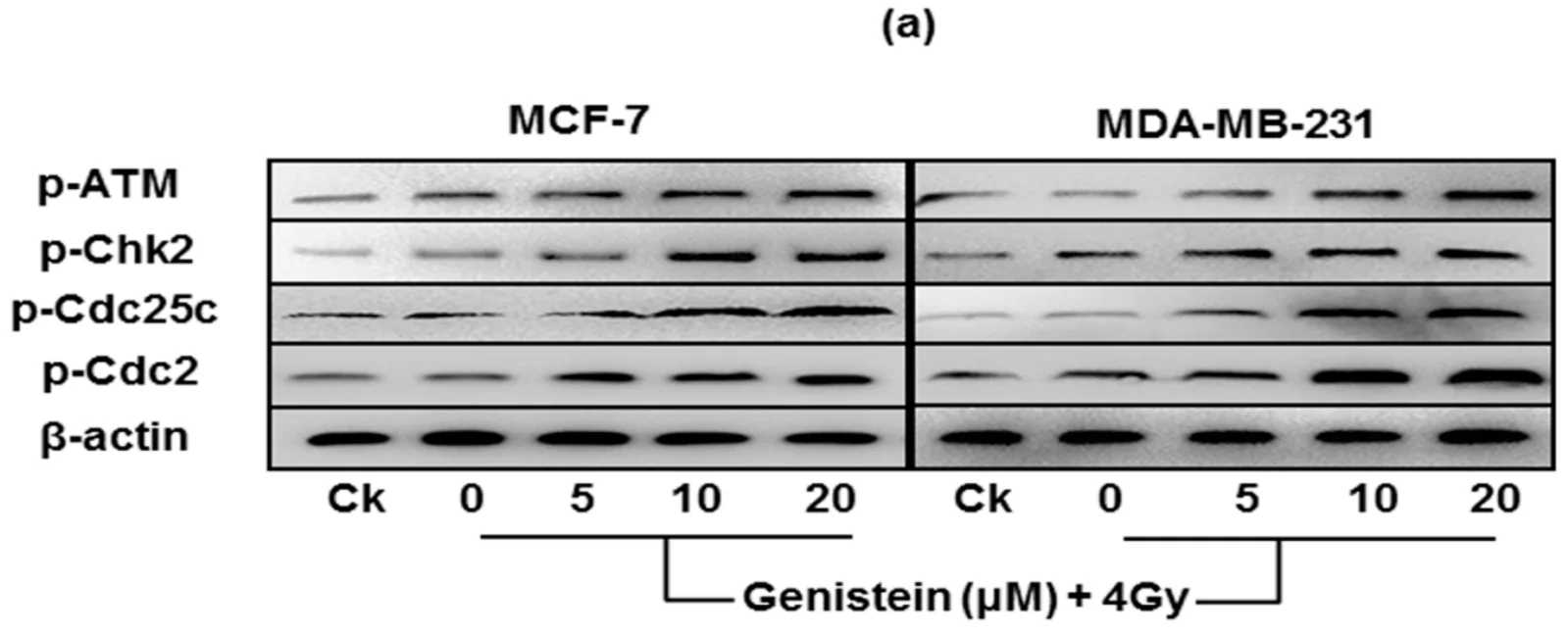

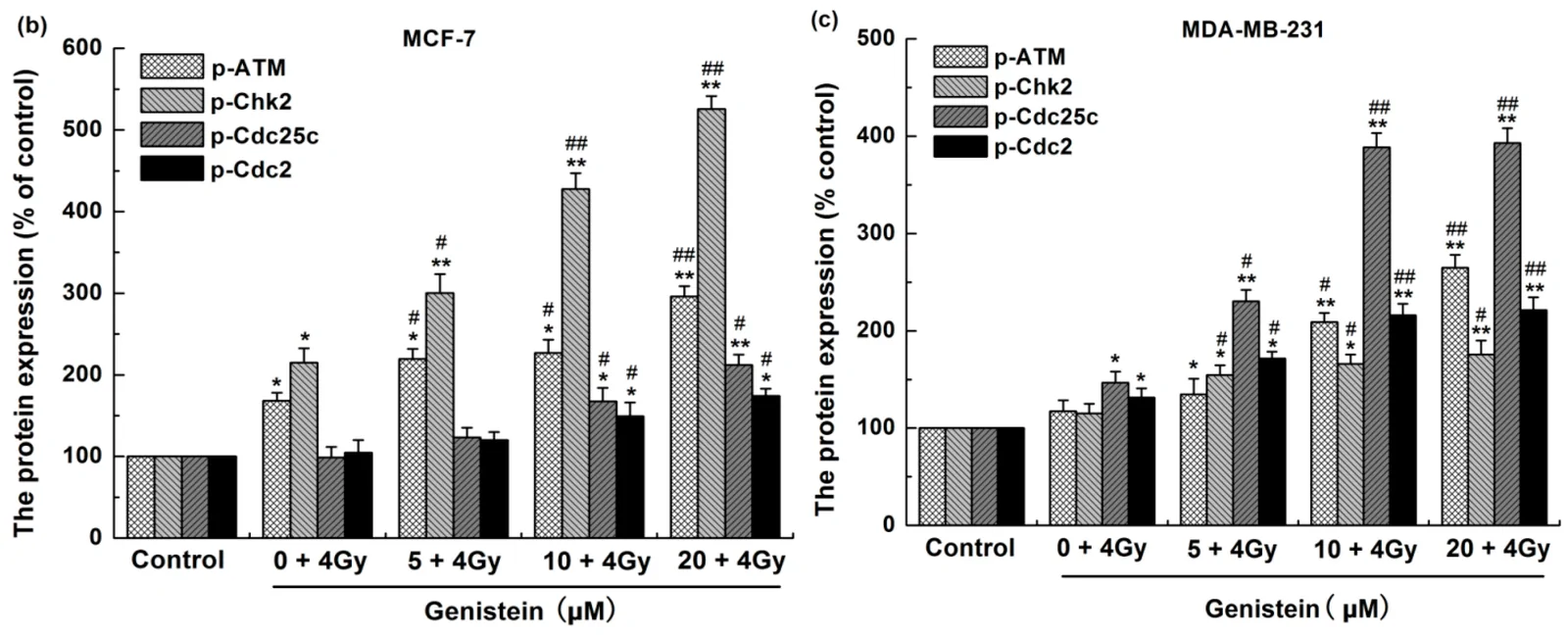
Effect of genistein combined with X-ray irradiation on the expression of G_2_/M checkpoint proteins in MCF-7 and MDA-MB-231 cells. (a) Representative blots from three independent experiments, where β-actin served as a loading control; (b) Quantative protein expression levels relative to control in MCF-7 cells; (c) Quantative protein expression levels relative to control in MDA-MB-231 cells. All data are presented as means ± SD from three independent experiments. * *p* < 0.05, ** *p* < 0.01 *versus* control group;^ # ^*p* < 0.05, ^## ^*p* < 0.01 *versus* X-rays alone.

Shown in Figure 8 are the expression levels of cell-cycle-related proteins in MCF-7 and MDA-MB-231 cells under the various conditions, as detected by western blot analysis. Clearly, in both cell lines, the level of pATM-Ser1981 was up-regulated, indicating that DNA damage checkpoint response was activated. As an indicator of cell-cycle checkpoint, the level of pChk2-Thr68 was also increased. Furthermore, the levels of pCdc25c-Ser216 and pCdc2-Tyr15 were markedly increased, demonstrating that in response to DNA damage, cells underwent the phosphorylation and inactivation of Cdc25c and Cdc2. Once the Cdc2 was inactivated, the DNA-damaged cells could not enter into mitosis and were arrested at G_2_/M phase.

**Figure 9:**
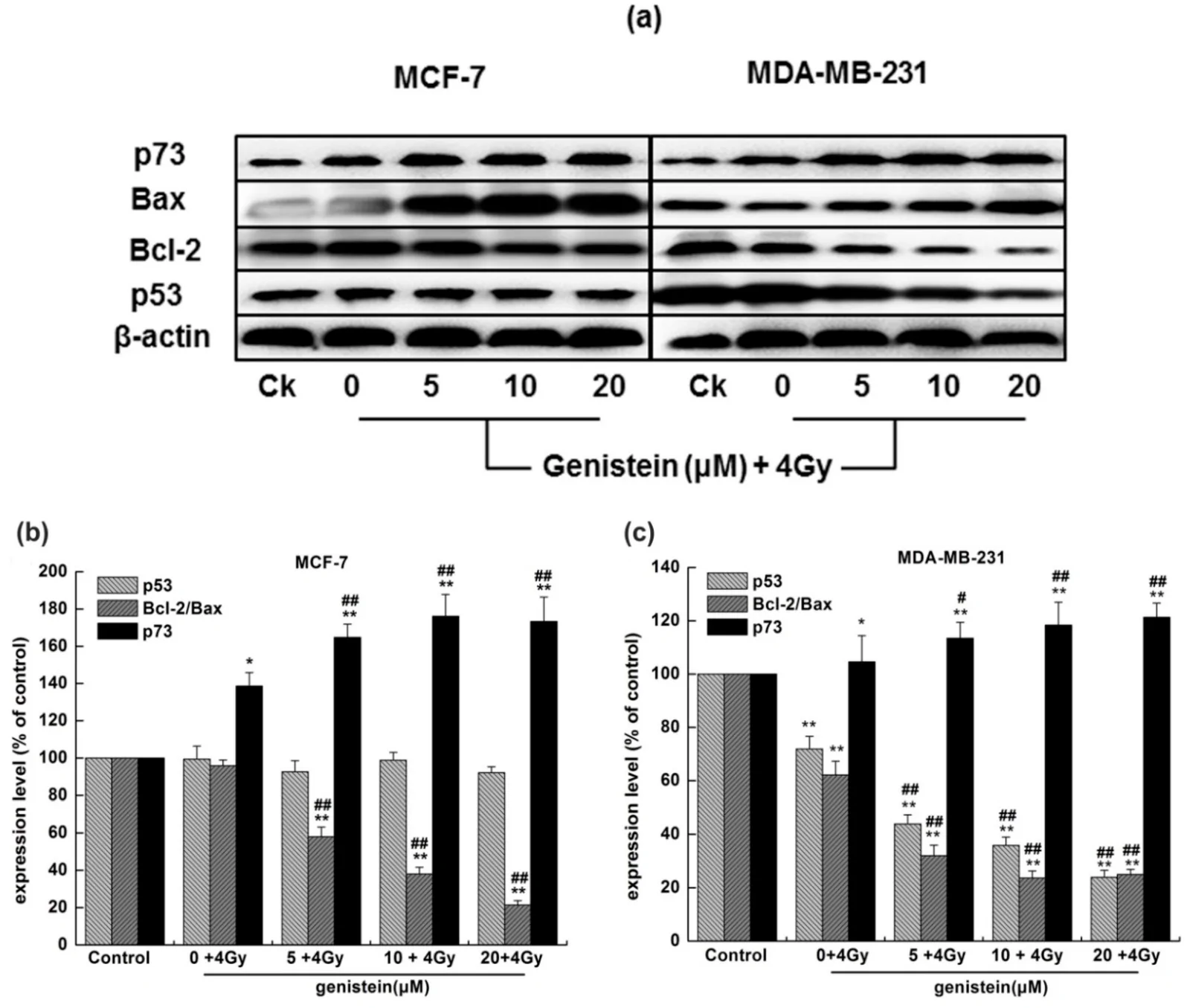
Effect of genistein combined with X-ray irradiation on the expression of cell apoptosis-related proteins in MCF-7 and MDA-MB-231 cells. (a) Representative blots from three independent experiments, where β-actin served as a loading control; (b) Quantative protein expression levels relative to control in MCF-7 cells; (c) Quantative protein expression levels relative to control in MDA-MB-231 cells. All data are presented as means ± SD from three independent experiments. * *p* < 0.05, ** *p* < 0.01 *versus* control group; ^# ^*p* < 0.05, ^## ^*p* < 0.01 *versus* X-rays alone.

To understand the effect of the molecular events involved in combined treatment on cell apoptosis, we next investigated the expression of p53, Bcl-2 and Bax, which are pivotal for cell apoptosis. Shown in Figure 9 are their expressions in MCF-7 and MDA-MB-231 cells. Clearly, there were two different modes of p53 expression in both cell lines. The combined treatment barely changed the expression of p53 in MCF-7 cells, but the mutant p53 expression in MDA-MB-231 cells was down-regulated at the protein level. Meanwhile, up-regulation of Bax and down-regulation of Bcl-2 were observed, suggesting the decrease of Bcl-2/Bax ratios might be involved in apoptosis induced by genistein combined with X-rays in both breast cancer cell lines. Additionally, the expression of p73, which is a member of the p53, family changed as well [21]. Western blot analyses show that the expression of p73 proteins was up-regulated significantly in both MCF-7 and MDA-MB-231 cells after the combined treatment (Figure 9). These results indicate that increase of p73 protein translation might result in the induction of apoptosis in both cell lines.

### 2.8. Discussion

Cancer has been the leading global causes of death, and the cancer mortality continues to rise every year. Radiotherapy (RT) is one of the most important therapeutic approaches for malignant diseases, and about 70% cancer patients will be treated with RT. However, the serious cytotoxicity to normal tissues and tumor radioresistance are two fatal flaws in RT. Fortunately, researchers have found that some natural compounds such as isoflavone, isoliquiritigenin and parthenolide have both hypotoxicity and radiosensitization characteristics [22,23,24].

Genistein is a phytoestrogen, present in legumes and other plants, and has been the subject of considerable attention due to its beneficial effects on chronic diseases including breast cancer [25]. There have been reports on the toxicity of genistein. Li *et al.* [26] showed that concentrations of genistein in the 5–15 μM range were considered mild doses and had no toxic effects on normal breast epithelial cells *in vitro*. We demonstrated that concentration of genistein at 5–20 μM had little toxicity to both MCF-7 and MDA-MB-231 cells *in vitro.* This is consistent with the previously reported result, demonstrating that the concentrations of genistein used in our study were safe. Meanwhile, Wang *et al* [27] demonstrated that the effect of genistein on cell growth at lower concentrations appeared to be via the estrogen receptor pathway, while the effect of genistein at higher concentrations (10 μM), was independent of the estrogen receptor. Zava and Duwe [28] found that in ER+ cell lines, the cell growth was not inhibited by 10 nM–10 μM of genistein, but abruptly inhibited by 20 μM genistein. In ER- breast cancer cell lines, genistein had little effect at 10 nM–1 μM. At concentrations greater than 1 μM, cell growth was markedly inhibited by genistein in ER-cell lines, similar to the effect seen in ER+ cell lines. However, our findings indicate that the response of MCF-7 and MDA-MB-231 cells to genistein was concentration-dependent, but independent from the ER status. Compared with the results derived from the X-ray irradiation alone group, with the increase of genistein concentration, a reduced clonogenic survival fraction, increased apoptotic rates and γ-H2AX foci numbers were observed in the combined-treatment groups (Figure 3 and Figure 4). Our data definitely indicate that genistein obviously enhanced the radiosensitivity of breast cancer MCF-7 and MDA-MB-231 cells instead of the direct toxicity by itself to cancer cells.

It was well known that the growth and proliferation of mammalian cells are mediated via cell cycle progression. In recent years, studies have shown an association between cell cycle regulation and cancer, and inhibition of the cell cycle has become an appreciated target for cancer management [15]. As shown in Figure 5, when the cells were pretreated with 5, 10 and 20 μM genistein for 24 h, the numbers of MCF-7 and MDA-MB-231 cells at G_2_/M phase were both increased in a genistein concentration-dependent manner. Therefore, the G_2_/M arrest induced by genistein might enhance the radiosensitivity in both cell lines. As shown in Figure 6(a), the combined treatment obviously augmented the number of cells at G_2_/M phase, compared with the genistein treatment alone group (Figure 5). However, with the time lapse post-irradiation, the number of cells at G_2_/M phase decreased sharply (see Figure 6(b)). Classical theories considered that the principal biological task of the cell cycle arrest is to allow sufficient time for repairing DNA damages. If the repair is completed, the cells can resume the cell cycle. Once the DNA damages are too serious to be repaired, the cells will ultimately die [29,30]. As shown in Figure 7, co-localization of γ-H2AX and Rad51 foci indicated that genistein treatment could block the recruitment of Rad51 to the injured sites of DNA and the apoptotic rates increased remarkably from 12 h to 24 h post-irradiation. These data illustrated that genistein pretreatment exacerbated the G_2_/M arrest at 12 h post-irradiation (Figure 6(a)), and a majority of the damaged cells in G_2_/M phase could not be repaired through the homology recombination mechanism (Figure 7(a)), but underwent death in an apoptosis-related manner (Figure 7(c,e)). The results definitely suggest that genistein-exacerbated G_2_/M arrest and subsequent apoptosis-related cell death are two main factors for enhancing the radiosensitivity of both MCF-7 and MDA-MB-231 cell lines.

Cell cycle checkpoint proteins play an important role in the cell cycle progress. As shown in Figure 8, the pretreatment with genistein followed by 4 Gy X-ray irradiation significantly induced the activation of ATM in both cell lines, indicating that ATM was actively involved in the response to DNA damage and G_2_/M arrest. Moreover, the levels of Chk2-Thr68, Cdc25c-Ser216 and Cdc2-Tyr15 increased by the combined treatment (see Figure 8). This demonstrates that activated ATM phosphorylated checkpoint proteins carried out the cellular responses upon genomic stress and subsequently activated downstream signal transducers.

Besides DNA damage and G_2_/M phase arrest, apoptosis was clearly seen in the breast cancer cells under investigation after genistein pretreatment combined with X-ray irradiation. The pro- and anti-apoptotic proteins of the Bcl-2 family may turn on and off apoptosis because of the formation of heterodimers among these proteins [31]. The heterodimerization results in mutual neutralization of the bound pro-and anti-apoptotic proteins. Therefore, the balance between the expression levels of the protein units (e.g. Bcl-2 and Bax) is critical for cell survival or death. Our result shows that the combined treatment led to up-regulation of Bax (a pro-apoptotic protein) level and concomitantly down-regulation of Bcl-2 (an anti-apoptotic protein) level in both cell lines (Figure 9(a)), and the Bcl-2/Bax rate decreased significantly compared with that in the cells exposed to X-rays alone (Figure 9(b, c)).

The wild-type p53 protein is known for its ability to induce apoptosis, the most important anti-tumor barrier [32,33]. However, p53 is the most frequently mutated gene in human cancers (more than 50%), and the mutant p53 loses its ability to inhibit cell growth. In some ways, it can inhibit cell apoptosis and induce carcinogenesis after exposure to DNA-damaging agents [13,34]. The results of our study definitely show that there was almost no change in p53 expression in MCF-7 cells (harboring wild-type p53) after the combined treatment while the mutant p53 expression was down-regulated remarkably in MDA-MB-231 cells (harboring mutant p53) (Figure 9). p73 is one member of the p53 gene family [35] , which has more than 60% amino acid identity within the DNA binding region of p53. As a result, a large number of p53 target genes are also trans-activated by p73. Hence, p73 shares some functions of p53 such as inducing apoptosis [21,36]. As shown in Figure 9, the expression of p73 increased obviously in both cell lines after genistein treatment combined with X-ray irradiation. Therefore, our study indicates that the combined treatment induced apoptosis in the breast cancer cells through the mitochondrial pathway, probably via the p73 mediated manner.

The present study has proved that genistein has radiosensitization efficacy on MCF-7 and MDA-MB-231 breast cancer cell lines. Interestingly, Nguyen *et al.* [37] found that genistein selectively induced growth arrest and G_2_/M phase cell cycle block in T47D tumorigenic breast epithelial cells but not normal breast epithelial cells (MCF-10A), and they believed that the important metabolic differences exist between tumorigenic and nontumorigenic cell lines. Besides, the work by Leung *et al.* [38] also proved that genistein protected MCF-10A cells against polycyclic aromatic hydrocarbon-induced oxidative DNA damage. Our data together with these results published previously suggest that genistein possibly possesses potent selective radiosensitization activity on cancer cells. Therefore, we propose that genistein might be an effective constituent of radiosensitization products.

## 3. Experimental

### 3.1. Cell Culture and Genistein Treatment

Established human breast cancer MCF-7 and MDA-MB-231 cell lines were purchased from the Type Culture Collection of the Chinese Academy of Sciences (Shanghai, China), and preserved in our laboratory. MDA-MB-231 and MCF-7 cells were maintained in Dulbecco's Modified Eagle's Medium (DMEM, Gibco, Grand Island, NE, USA) supplemented with 100 units/ml penicillin, 100 μg/ml streptomycin (Gibco) and 10% fetal bovine serum (FBS, Biowest, Nuaillé, France). All cells were cultured at 37 °C in a humidified atmosphere containing 5% CO_2_. Genistein (Sigma, St. Louis, MO, USA) was dissolved in dimethyl sulfoxide (DMSO, Sigma), the stocked solution was 15 mM in DMSO and stored at −20 °C. 50%–60% confiuent cells were treated with genistein, where the concentrations of DMSO were less than 0.5% in all experiments.

### 3.2. Irradiation

After the desired times of genistein pretreatment, cells were irradiated with X-rays, which were generated with an X-ray machine (FAXITRON RX-650, Faxitron Bioptics, LLC, Tucson, AZ, USA) operated at 100 kVp energy. The dose rate was about 1 Gy/min. All irradiations were carried out at room temperature.

### 3.3. MTT Proliferation Assay

The inhibition of cell proliferation by genistein was determined by use of the MTT assay [39], monitoring the number of cells based on the reduction of MTT by the mitochondrial dehydrogenase presented in viable cells. Briefly, cells were plated into 96-well tissue culture dishes at a density of 1 × 10^4 ^cells/well in 180 µL medium. After plating, the cells were allowed to attach for 24 h then added genistein. Incubation with genistein continued for 48 h, at which time 20 µL of MTT (5 mg/mL) was added to each well. After incubation at 37 °C for 4 h, the supernatants were removed and formazan crystals were dissolved by adding 150 µL DMSO. The well plates were then read on a microplate reader at 490 nm. The reduction in viability of genistein-treated cells was expressed as a percentage compared to non-genistein (DMSO) treated cells and control cells were considered to be 100% viable. Each experiment was conducted in triplicate.

### 3.4. Colony Formation Assay

Cell survival was measured with the standard colony forming assay. Briefly, cells were pretreated with genistein for 24 h followed by X-rays. Immediately after irradiation, the cells were collected by trypsinization, diluted and seeded in 100 mm Petri dishes. The dishes were cultured for 15 days in DMEM without genistein, then fixed with alcohol and stained with crystal violet. Colonies containing more than 50 cells were identified as survivors under a stereomicroscope. Each experiment was performed in triplicate.

### 3.5. γ-H2AX and Rad51 Foci Immunofluorescence

Cells were plated at a density of 1 × 10^5^ cells/well into a 6-well plate covered by a coverslip and allowed to attach for 24 h. Then the cells were pretreated with different concentrations of genistein for 24 h followed by 4 Gy X-rays. At 12 h post-irradiation, the cells were fixed with 4% paraformaldehyde for 10 min. The fixed cells were permeabilized with 0.1% Triton X-100 and subsequently blocked with 5% BSA at room temperature for 1 h. The cells were then incubated overnight at 4 °C with primary monoclonal antibodies against γ-H2AX (mouse monoclonal to γ-H2AX) and Rad51 (rabbit monoclonal to Rad51) (Abcam, Cambridge, MA, USA, 1:200 dilution). After primary antibody incubation, the cells were washed with PBST and incubated with donkey anti-mouse IgG-FITC secondary antibody (Figure 4), rabbit anti-mouse IgG-R secondary antibody (Figure 7(a), immunofluorescence for γ-H2AX) and donkey anti-rabbit IgG-FITC secondary antibody (Figure 7(a), immunofluorescence for Rad51) (Santa Cruz, Dallas, TX, USA, 1:300 dilution) for 1 h at room temperature. Nuclei were counterstained with mounting medium DAPI (1.5 μg/mL, VECTASHIELD Mounting Medium, Vector Lab, Inc., Burlingame, CA, USA). Finally, γ-H2AX and Rad51 foci were detected with a laser scanning confocal microscope (LSM 700, Zeiss, Oberkochen, Germany).

### 3.6. Cell Cycle Progression Analysis

The protocol was performed as previously described by Vindelov *et al* [40]. When harvesting, cells were washed with PBS (phosphate buffered saline) prior to and following trypsinization, then fixed in 75% pre-chilled ethanol for over 48 h at −20 °C. The fixed cells were spun down and resuspended in PBS at a concentration of 1 × 10^6^ cells/mL, then 50 μL of 100 μg/mL RNase (Sigma) was added. The cells were incubated for 30 min with RNase solution and then put on ice. Immediately prior to measurement, the cells were stained by adding 100 μl of 50 μg/mL PI (propidium iodide, Sigma). Cell cycle distribution was analyzed with the cell-cycle data analysis software (FlowJo 7.6.1) from the histogram of DNA content measured with a flow cytometer (FACScan, Becton Dickinson, city, CA, USA). Unstained cells were analyzed as controls and used to gate on live cells for final analysis.

### 3.7. Detection of Apoptosis

Apoptosis was quantified by a combined staining of Annexin V and PI using Annexin V-FITC Apoptosis Detection Kit (BestBio, Shanghai, China). Annexin V-FITC was used to probe the appearance of PS (phosphotidylserine) residues on the outer leaflet of the plasma membrane as previously described [41]. Cells were prepared according to the manufacturer’s instructions. Briefly, approximately 1 × 10^6 ^cells per experimental condition were harvested, washed with cold PBS twice, and resuspended with 400 µL binding buffer. After adding 5 μL of Annexin V-FITC solution and 10 μL of PI solution, the cells were incubated for 15 min at room temperature in the dark. After the incubation, 10,000 cells were analyzed with the flow cytometer.

### 3.8. Western Blot Analysis

Western blot analysis was performed following the standard methods. Briefly, cell lysates were resolved by SDS-PAGE under reducing conditions at a concentration of 20 μg protein of each sample per lane and then transferred to PVDF membranes (Millipore, Bedford, MA, USA). Blots were blocked with 5% nonfat milk in TBST for 1 h and then incubated overnight with primary antibodies (anti-pATM, anti-pChk2, anti-pCdc25c and anti-pCdc2, anti-p53, anti-p73, anti-Bcl-2, anti-Bax 1:500 dilution, Santa Cruz). They were then washed and incubated with a secondary peroxidase-conjugated antibody (1: 2000 dilution, Abcam). Bound secondary antibody was detected using a chemiluminescence (ECL, Roche, Shanghai, China) system according to the manufacturer’s protocol. To confirm equal protein loading per lane, the membranes were subsequently reprobed with a 1:1,000 dilution of an anti-β-actin antibody (Abcam) and developed as described above.

### 3.9. Statistical Analysis

The data were obtained from three independent experiments and are presented as means ± SD (standard deviations). Statistical comparisons of the mean values were performed using the Student’s t-test. Differences with a *p*-value of less than 0.05 are considered statistically significant.

## 4. Conclusions

This study has shown that the response of breast cancer cells to genistein is concentration-dependent but independent from ER and p53 type. Genistein has the function to enhance the radiosensitivity of breast cancer cells regardless of ER status, which is closely associated with the induction of G_2_/M arrest and subsequent apoptosis through the mitochondrial-mediated pathway. To attain better therapeutic outcome in breast cancers, radiotherapy combined with genistein seems to be a potential modality. Further studies to assess the radiosensitization effect of genistein *in vivo* are in progress.

## Abbreviations

ER: estrogen receptor
ER+: estrogen receptor positive
ER-: estrogen receptor negative
Genistein: 5,7,4-trihydroxyisoflavone
DMEM: Dulbecco’s Modified Eagle’s Medium
DMSO: dimethyl sulfoxide
γ-H2AX: phosphorylation of histone H2AX
DSB: DNA double-strand breaks
PBS: phosphate buffered saline
PI: propidium iodide
RT: Radiotherapy
