# Correction: Liu et al. Genistein Enhances the Radiosensitivity of Breast Cancer Cells via G_2_/M Cell Cycle Arrest and Apoptosis. *Molecules* 2013, *18*, 13200–13217

**DOI:** 10.3390/molecules31203646

**Published:** 2026-10-13

**Authors:** Xiongxiong Liu, Chao Sun, Xiaodong Jin, Ping Li, Fei Ye, Ting Zhao, Li Gong, Qiang Li

**Affiliations:** 1 Institute of Modern Physics, Chinese Academy of Sciences, Lanzhou 730000, China; lxx002@impcas.ac.cn (X.L.); sunchao@impcas.ac.cn (C.S.); jinxd@impcas.ac.cn (X.J.); liping@impcas.ac.cn (P.L.); yefei@impcas.ac.cn (F.Y.); zhaoting@impcas.ac.cn (T.Z.); gongli13@impcas.ac.cn (L.G.); 2 Key Laboratory of Heavy Ion Radiation Biology and Medicine, Chinese Academy of Sciences, Lanzhou 730000, China; 3 University of Chinese Academy of Sciences, Beijing 100049, China

In the original publication [1], there were mistakes in Figure 7 as published. Figure 7a contained a mismatch between the DAPI channel and corresponding Rad51 and γH2AX fluorescence signals in the MCF-7 4 Gy alone group. Figure 7b,c contained improper image layout. The flow cytometry data were re-analyzed as shown in Figure 7d,e. The corrected Figure 7 appears below.

A correction has been made to Section 2.6, in the last paragraph:

Next, we investigated whether genistein enhancement of the radiosensitivity of breast cancer cells was associated with cell apoptosis. Cells were pretreated with a range of genistein concentrations for 24 h, followed by 4 Gy X-rays. Figure 7b,c show the representative apoptosis results at 12 h and 24 h post-irradiation. At 12 h post-irradiation, the apoptotic rates were 21.9 ± 2.1% and 19.9 ± 2.7% in MCF-7 and MDA-MB-231 cells in the 20 μM genistein pretreatment group, in contrast to 8.9 ± 2.0% and 9.8 ± 2.4% in the control groups, respectively (Figure 7d). At 24 h post-irradiation, the apoptotic rate increased more significantly (Figure 7e).

The authors state that the scientific conclusions are unaffected. This correction was approved by the Academic Editor. The original publication has also been updated.

## Figures and Tables

**Figure 7:**
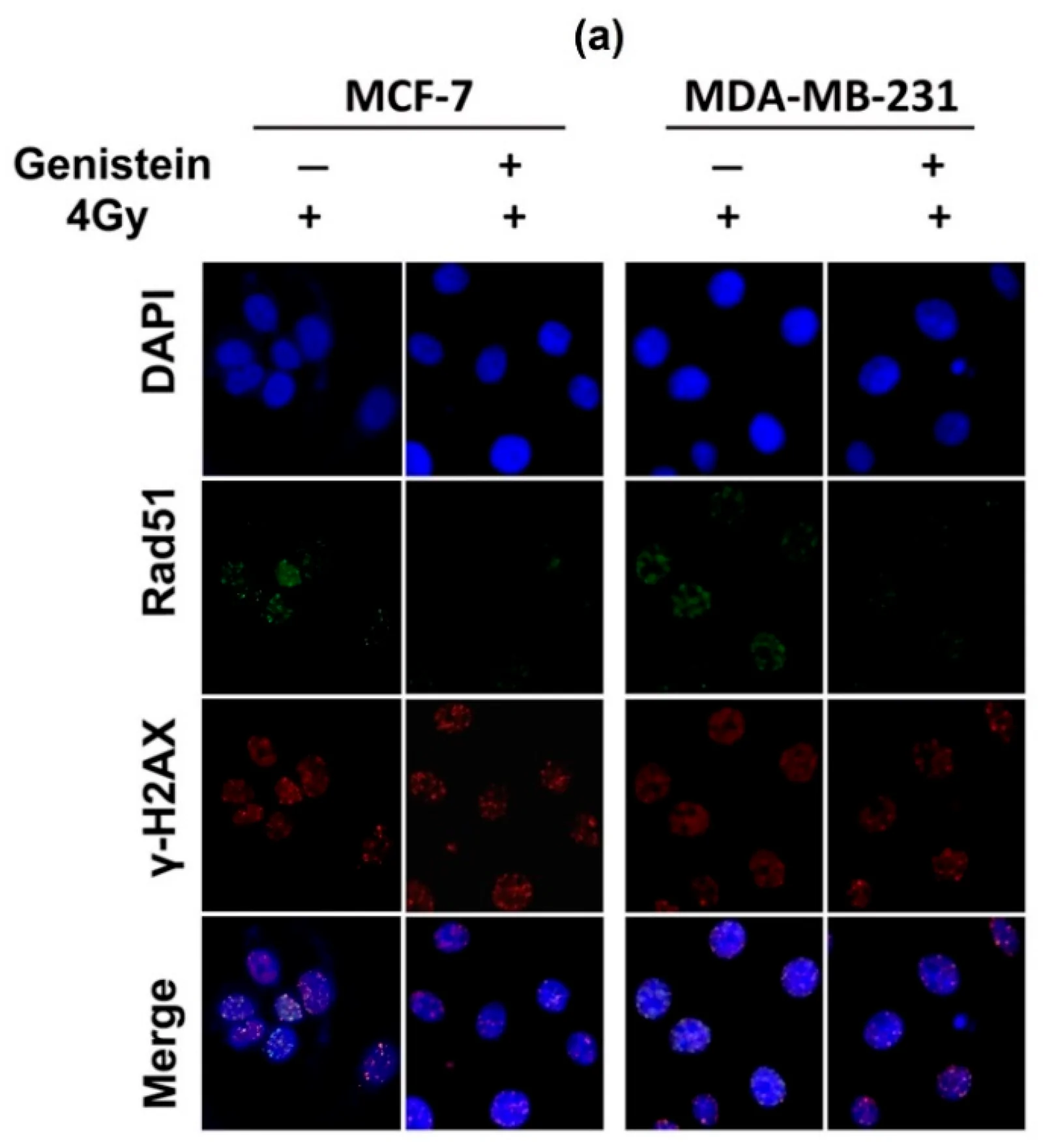

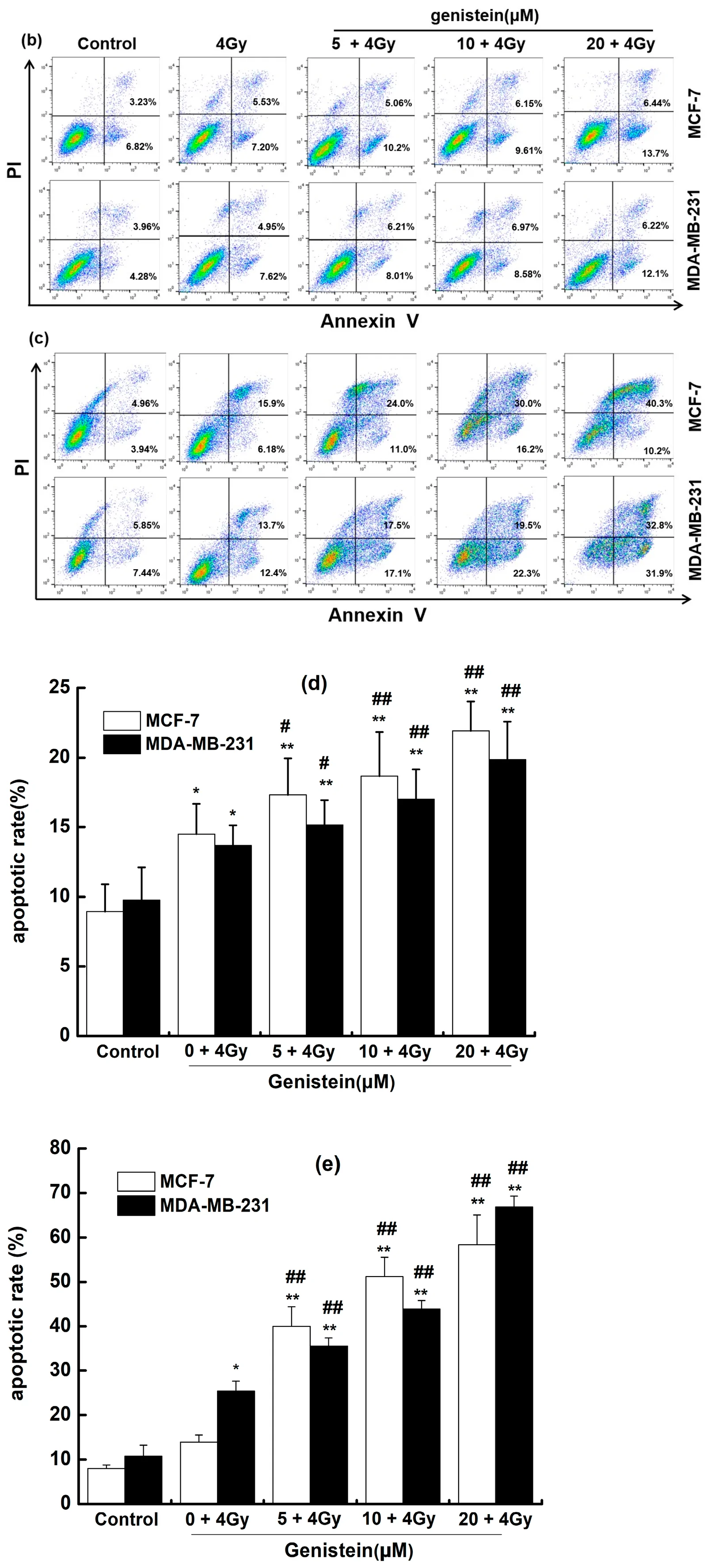
Effect of genistein combined with X-ray irradiation on the cell repair system and apoptosis of MCF-7 and MDA-MB-231 cells. (**a**) Co-localization of Rad51 (green points) and γ-H2AX (red points) foci; nuclear staining was done with DAPI (blue). Scale bars represent 20 μm; (**b**) Representative cell apoptosis of three independent experiments at 12 h post-irradiation; (**c**) Representative cell apoptosis of three independent experiments at 24 h post-irradiation; (**d**) Cell apoptotic rates at 12 h post-irradiation; (**e**) Cell apoptotic rates at 24 h post-irradiation. All data are presented as means ± SD from three independent experiments. * *p* < 0.05, ** *p* < 0.01 versus control group; ^#^ *p* < 0.05, ^##^ *p* < 0.01 versus X-rays alone.
